# Combined surgical-orthodontic treatment of patients with cleidocranial dysplasia: case report and review of the literature

**DOI:** 10.1186/s13023-018-0959-3

**Published:** 2018-12-04

**Authors:** Yanfei Zhu, Yin Zou, Qian Yu, Huijun Sun, Sixuan Mou, Shuhua Xu, Min Zhu

**Affiliations:** 1 Department of Oral and Craniomaxillofacial Science, Shanghai Ninth People’s Hospital, College of Stomatology, Shanghai Jiao Tong University School of Medicine, No 500, Quxi Road, Shanghai, Shanghai, China; 2 Center for Specialty Strategy Research of Shanghai Jiao Tong University China Hospital Development Institute, Shanghai200011, Shanghai, China; 3 Department of Stomatology, Wuxi People’s Hospital Affiliated to Nanjing Medical University, No 299 Qingyang Road, Wuxi214000, Jiangsu, China; 4 Shanghai Key Laboratory of Stomatology & Shanghai Research Institute of Stomatology, National Clinical Research Center for Oral Diseases, Shanghai200011, Shanghai, China

**Keywords:** Cleidocranial dysplasia, Surgical-orthodontic treatment, Systematic review, Case report

## Abstract

**Objectives:**

To study the present treatment situation and investigate a better orthodontic approach for patients with cleidocranial dysplasia (CCD) through systematically reviewing the published cases and to conclude the surgical-orthodontic treatment experience of cleidocranial dysplasia.

**Methods:**

A comprehensive search for studies published through to April 10, 2018 was conducted using the Pubmed, Web of Science, and Embase databases. The CCD cases treated with the approach combining surgical exposure and orthodontic treatment were concluded.

**Results:**

Eight papers and 9 finished cases were included to be compared with the present case. The age of cases ranged from 9 to 28 years. Clearing the way of eruption path in early age can facilitate the spontaneous eruption of impacted teeth. For adults, combined surgical-orthodontic treatment can achieve a nearly complete dentition and stable occlusal contact, but it is time consuming and needs surgical assistance. The combination of orthognathic surgery can reduce the difficulty of orthodontic treatment and treatment duration, as well as achieve a better facial profile.

**Conclusion:**

Surgical exposure combined with orthodontic traction is an effective treatment for patient with CCD. Patient’s age, demand, economic circumstances, and status of permanent dentition should be considered when making treatment plan.

## Background

Cleidocranial dysplasia (CCD) was first named by Marie and Sainton in 1897 with the characteristics of aplastic or hypoplastic clavicles, exaggerated development of the transverse diameter of cranium, and delayed ossification of the skull. The prevalence of CCD is one per million with equal frequency among males and females [1]. This syndrome is an autosomal dominantly inherited disease with skeletal dysplasia caused by mutations in RUNX2, an osteoblast-specific transcription factor-encoding gene situated in the chromosomal locus of 6p21 [2].

### Manifestations

CCD prominently affects bones of membranous origin. Affected individuals typically present characteristics of short stature, long appearance of the neck and markedly slopping shoulders [3]. A narrow thorax allows the proximity of the shoulders in front of the chest [4]. Delayed closure of the pubic symphysis, coxa vara, or coxa valga [5] and conduction hearing impediment [6] have also been described. The mental development of these patients is usually normal.

Facial and cranium manifestations include delayed ossification of the skull, brachycephalic head with an increased transverse diameter of cranium, pronounced frontal and parietal bone, occipital bossing, formation of Wormian bone, ocular hypertelorism, and broad-based nose [3, 4]. Patients tend to have a skeletal Class III malocclusion due to mandibular hyperplasia along with hypoplasia of mid-face. Vertical facial growth is decreased due to hypoplasia of alveolar bone [7].

Oral features include delayed exfoliation of primary teeth, multiple impacted permanent and supernumerary teeth combined with severe malocclusion and crossbite [8]. The permanent first and second molars are rarely affected, but spontaneous eruption is usually delayed [4]. Affected individuals are more likely to have cyst formation surrounding the impacted teeth. Teeth abnormalities include enamel and cementum hypoplasia, root dilaceration, and microdontia. Submucous cleft palate as well as complete cleft of hard and soft palates have also been reported [9].

### Management

The treatment alternatives of patients with CCD include prosthetic replacements, facilitation of unerupted permanent teeth through surgery by removing the overlying supernumerary teeth and bone, surgical exposure of impacted teeth combined with orthodontic treatment, and combined orthognathic-orthodontic treatment [10].

The four well-known surgical-orthodontic treatments are the Toronto-Melbourne, Belfast-Hamburg, Jerusalem, and Bronx approaches [11].

The Toronto-Melbourne approach is based on age. The best period for treatment begins at 5 to 6 years of age. The timing of serial extraction of primary teeth depends on the extent of the root lengths developed in permanent teeth. Supernumerary teeth are also extracted with the alveolar bone covering the impacted teeth. Rationale of this approach is to facilitate the spontaneous eruption of impacted permanent teeth, so there would be no need for orthodontic traction.

The Jerusalem approach needs 2 surgical procedures. In the first phase, the anterior primary teeth and all supernumerary teeth are extracted, followed by the exposure of permanent incisors at 10 to 12 years of age. In the second phase, the posterior primary teeth are extracted, and the impacted permanent canines and premolars are exposed after age of 13. The surgery removes the barrier on the eruption path and promotes the normal eruption pattern of impacted teeth. However, two-thirds of the roots in permanent teeth have already developed in this approach, further orthodontic traction is usually needed.

The Belfast-Hamburg approach is similar to Jerusalem approach, though the age is not specified. Only one surgery under general anesthesia is advocated to remove all primary and supernumerary teeth, and to expose the impacted permanent teeth. After healing orthodontic traction is performed.

In the Bronx approach, the first phase is to remove primary teeth as well as supernumerary teeth and expose the impacted teeth. The use of removable partial overdenture is for esthetic and functional purposes. Orthodontic treatment starts after the spontaneous eruption of permanent teeth for sufficient posterior support, then a Le Forte I osteotomy is performed. Finally implants are placed to restore dentition defect.

Each approach has different indication and outcomes. The skeletal anomalies and complex multiple dentition of CCD add much difficulty and uncertainty to the orthodontic treatment. The treatment method of CCD is still under exploration. This study presents the management of combined surgical-orthodontic treatment in a Chinese female patient with CCD and summaries the approaches of orthodontic treatments for CCD through systematically reviewing the published cases.

## Case report

### Diagnosis and etiology

A 16–year–old female came for an orthodontic consultation in March, 2008 with chief complains of crossbite and failure of eruption of permanent dentition.

**Fig. 1:**
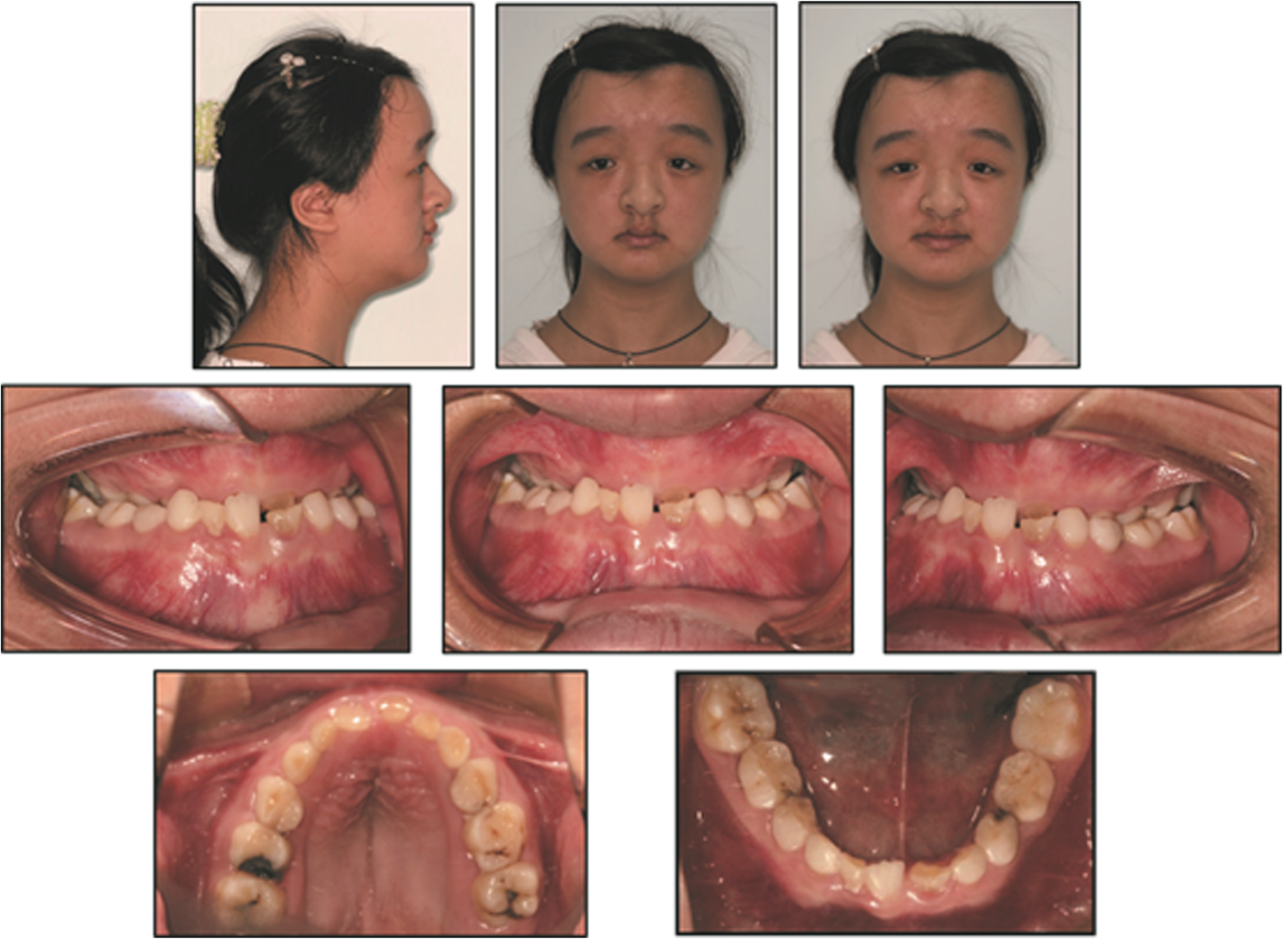
Pretreatment facial and intraoral photographs

Intraoral examination showed a mixed dentition with Class III malocclusion. The overjet was -3 mm, overbite was − 7 mm and the midlines were centered with no notable shift. Dental formula was as follows: (Fig. 1)

$$ \frac{6\kern0.24em \mathrm{V}\kern0.24em \mathrm{I}\mathrm{V}\kern0.24em \mathrm{I}\mathrm{I}\mathrm{I}\kern0.24em \mathrm{I}\mathrm{I}\kern0.24em \mathrm{I}\mid \mathrm{I}\kern0.5em \mathrm{I}\mathrm{I}\kern0.24em \mathrm{I}\mathrm{I}\mathrm{I}\kern0.24em \mathrm{I}\mathrm{V}\kern0.24em \mathrm{V}\kern0.24em 6}{6\kern0.24em \mathrm{V}\kern0.24em \mathrm{I}\mathrm{V}\kern0.24em \mathrm{I}\mathrm{I}\mathrm{I}\kern0.24em \mathrm{I}\mathrm{I}\kern0.24em \mathrm{I}\mid \mathrm{I}\kern1.5em \mathrm{I}\mathrm{I}\mathrm{I}\kern0.24em \mathrm{I}\mathrm{V}\kern0.24em \mathrm{V}\kern0.24em 6} $$

**Fig. 2:**
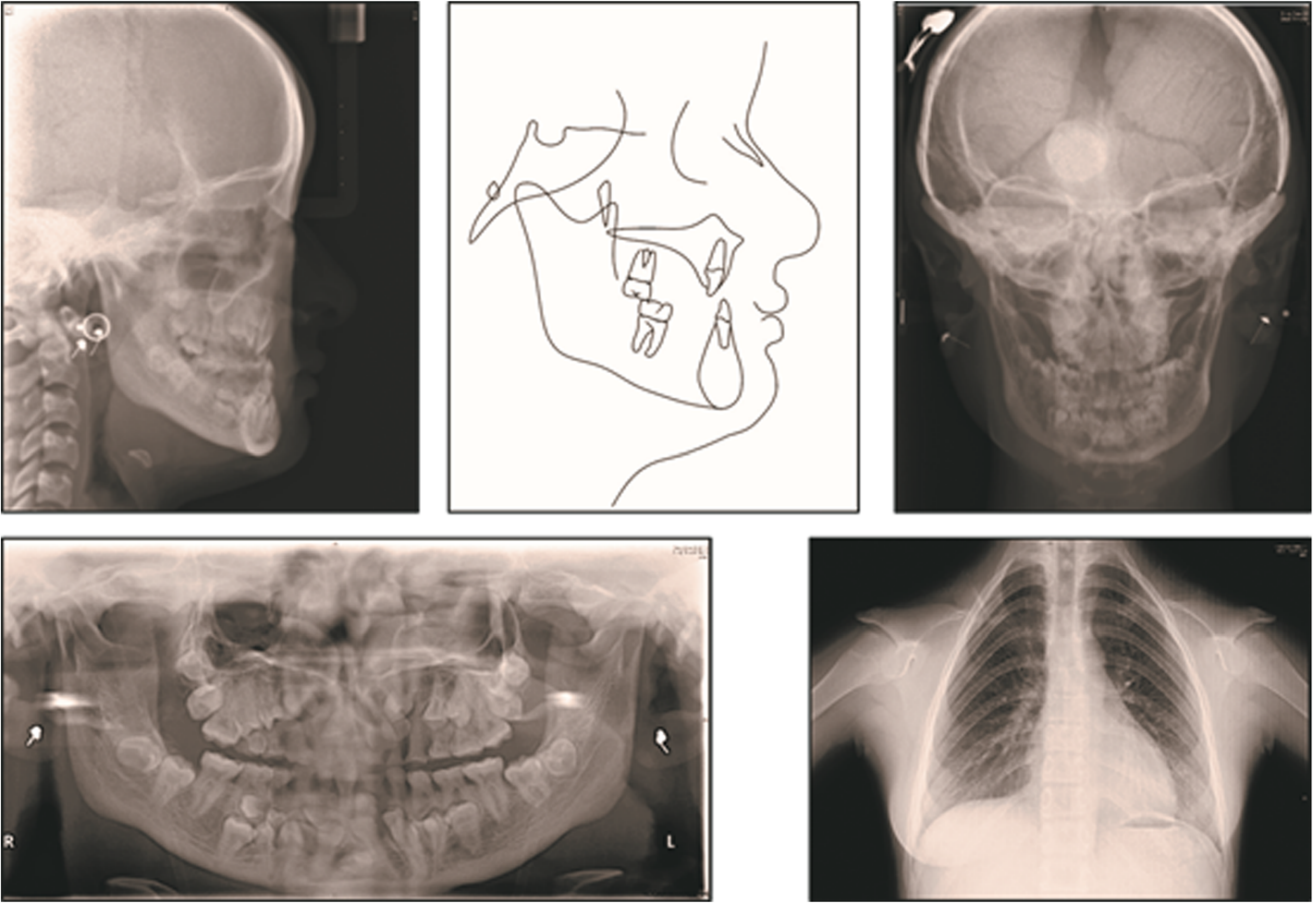
Pretreatment chest radiograph, panoramic radiograph, frontal radiograph, lateral radiograph, and cephalometric tracing

The panoramic radiograph revealed congenitally missing one lower incisor, the ectopic localization of permanent teeth, cysts formation involving the mandibular premolars, and the presence of 7 supernumerary teeth (1 in the maxilla and 6 in the mandible). The lateral radiograph confirmed a skeletal Class III malocclusion caused by mandibular hyperplasia and rotation with a horizontal growth (ANB = − 1°; Wits = − 0.3 mm; FMA = 20.2°). (Fig. 2).

This patient was diagnosed with cleidocranial dysplasia based on the presence of pathognomonic appearance, hypoplasia of clavicles, failure of permanent teeth eruption, and multiple supernumerary teeth.

### Treatment progress

**Fig. 3:**
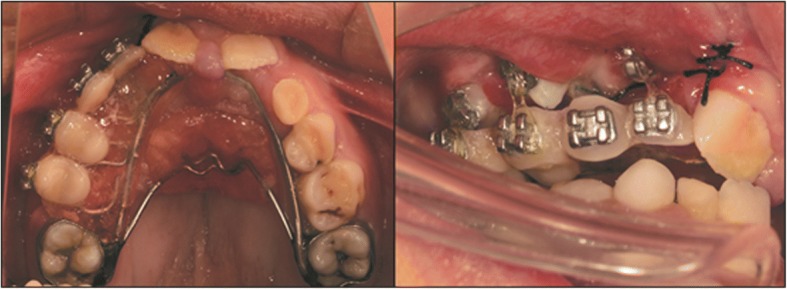
A dentomucosa-supported appliance for orthodontic traction

First all the primary teeth and the supernumerary teeth on the way of eruption path in maxilla were extracted. Impacted permanent teeth were surgically exposed under local anesthesia, followed by the immediate bonding of edgewise brackets to the exposed teeth surface for orthodontic traction. A dentomucosa-supported semi-fixed appliance with two bands bonded on the maxillary first molars was used as an anchorage for traction (Fig. 3). Artificial teeth with edgewise brackets were fixed on the resin base for traction and esthetics. Once the buccal surface of impacted teeth was exposed sufficiently, edgewise brackets were repositioned. Meanwhile, the same method was applied to guide mandibular teeth and brackets were bonded after traction. It took 5 years to expose all the impacted teeth and complete bonding of edgewise brackets. An orthodontic treatment was initiated after the exposure of all impacted teeth. Malligan expansion arch combined with Class III elastics was used to correct anterior and posterior crossbite.

### Treatment results

The treatment progress lasted for 8 years. For facial esthetics, a harmonious relationship of the facial soft tissues with normal convexity and a more pleasant smile were obtained by increasing the prominence of the upper lip.

**Fig. 4:**
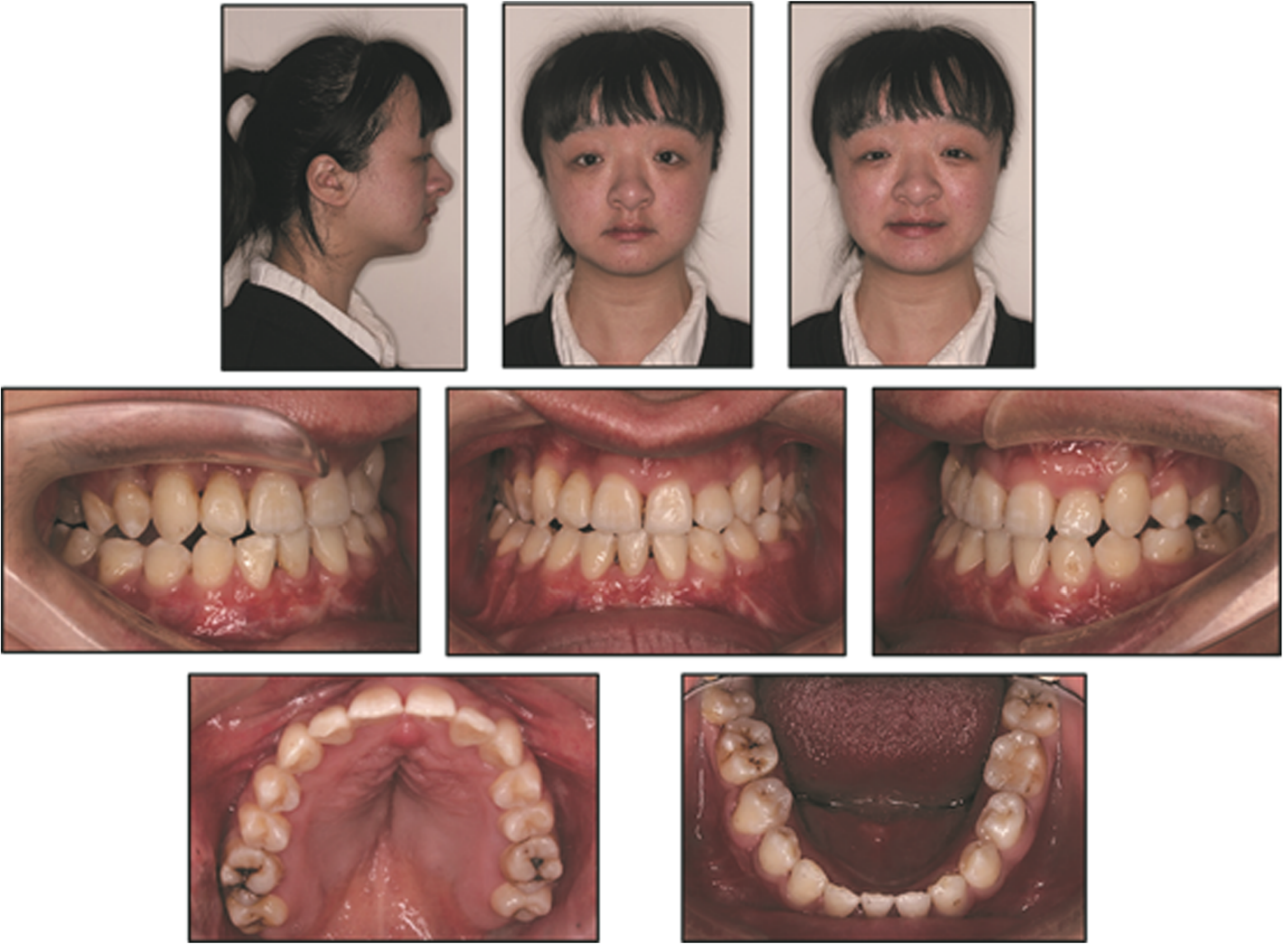
Post-treatment facial and intraoral photographs

**Fig. 5:**
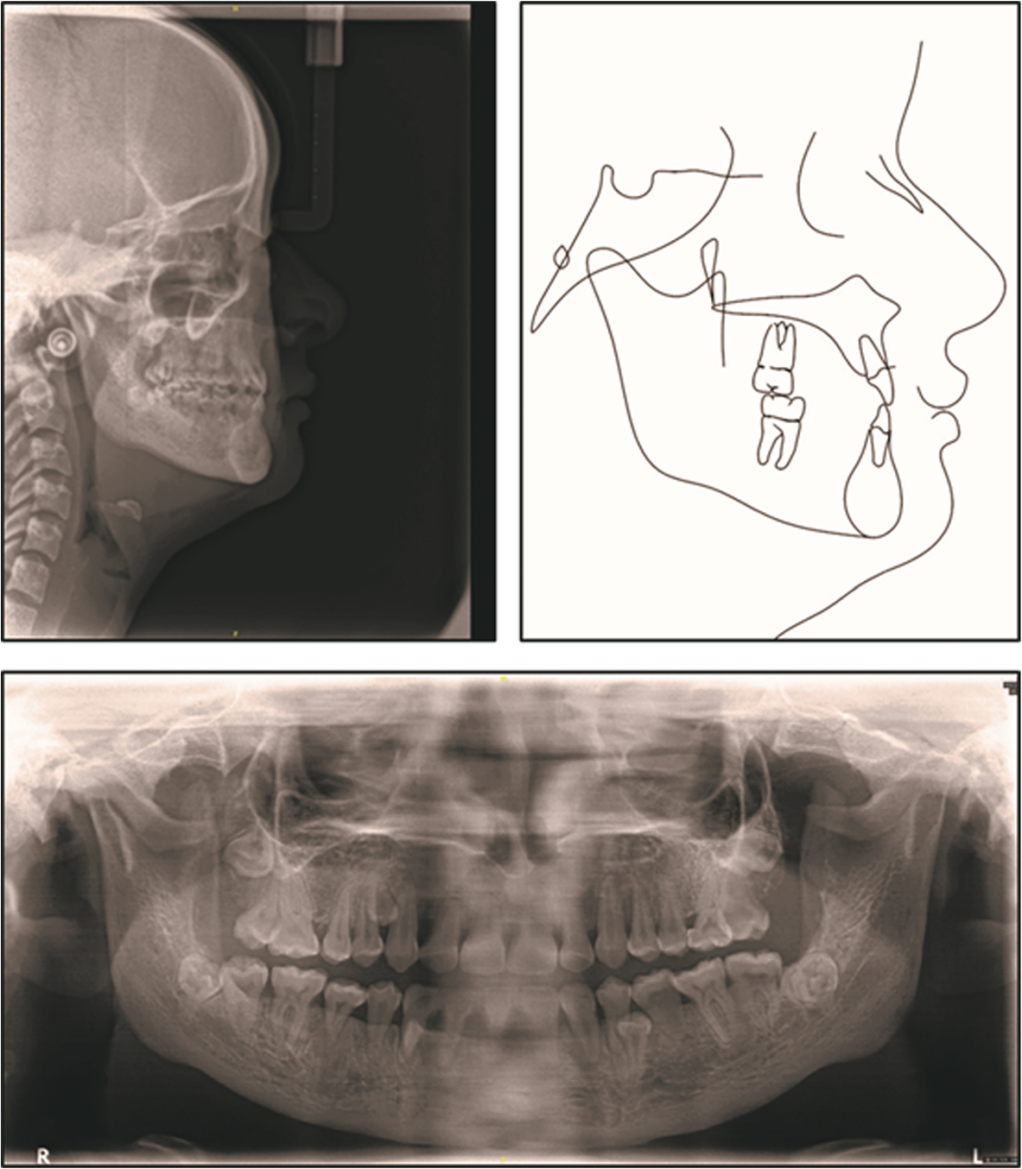
Post-treatment panoramic radiograph, lateral radiograph, and cephalometric tracing

Except the absence of one mandibular incisor, a complete dentition was obtained by orthodontic traction. The teeth were well positioned and a stable occlusion was established with normal overbite and overjet. Class III canine and molar relationships were finally achieved because of the absence of one mandibular incisor. (Fig. 4).Three supernumerary teeth which did not influence the alignment and stability of the dentition were left unextracted (Fig. 5).

**Fig. 6:**
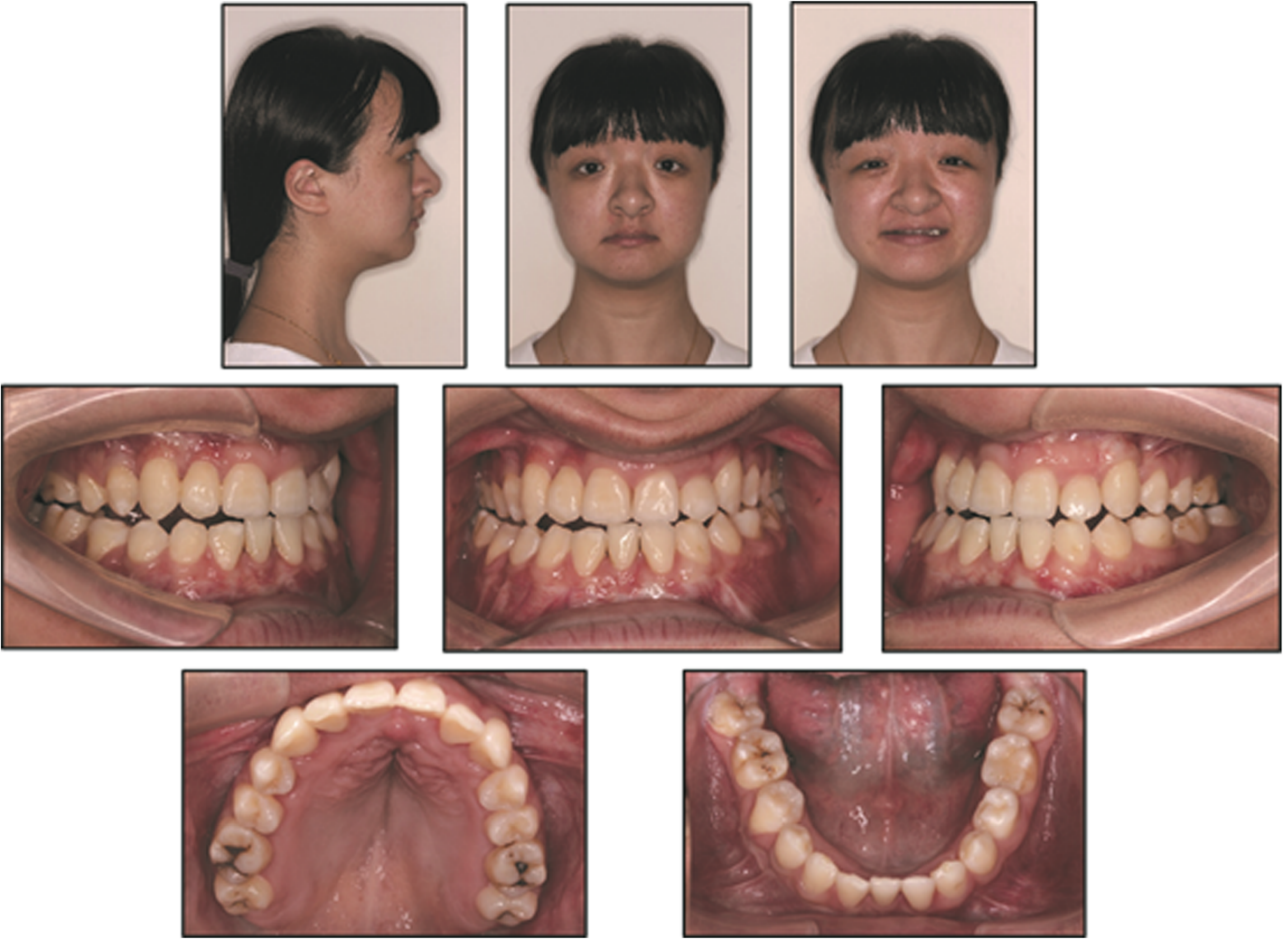
Facial and intraoral photographs at 1-year follow-up

After 1 year’s follow-up, the orthodontic results were relatively stable (Fig. 6).

## Systematic review of case reports

### Methods

A comprehensive search limited to English was conducted using the Pubmed, Web of Science, and Embase databases for studies published through to April 10, 2018. The inclusion criteria were as follows: (1) case report, (2) cases reporting the treatment of cleidocranial dysplasia, and (3) cases treated with the approach combining surgical exposure and orthodontic treatment. Furthermore, the exclusion criteria were: (1) uncompleted cases, (2) editorials, author opinions, or reviews. The search strategies were as follows: (case*) AND (Marie-Sainton syndrome OR cleidocranial dysplasia OR cleidocranial dysostosis) AND (orthodontic*). Two investigators screened the titles and abstracts separately for the selection of relevant cases. Cases that could not be excluded definitively on basis of the information gleamed from titles and abstracts were analyzed through full-texts. Disagreements would be resolved by a discussion held with a third investigator. The interreviewer reliability of study selection was evaluated by the percentage of agreement and value of Kappa.

The first author’ name, year of publication, patients’ basic information, treatment method and treatment duration were extracted from each paper. Qualitative results were extracted from the included studies.

### Results

**Fig. 7:**
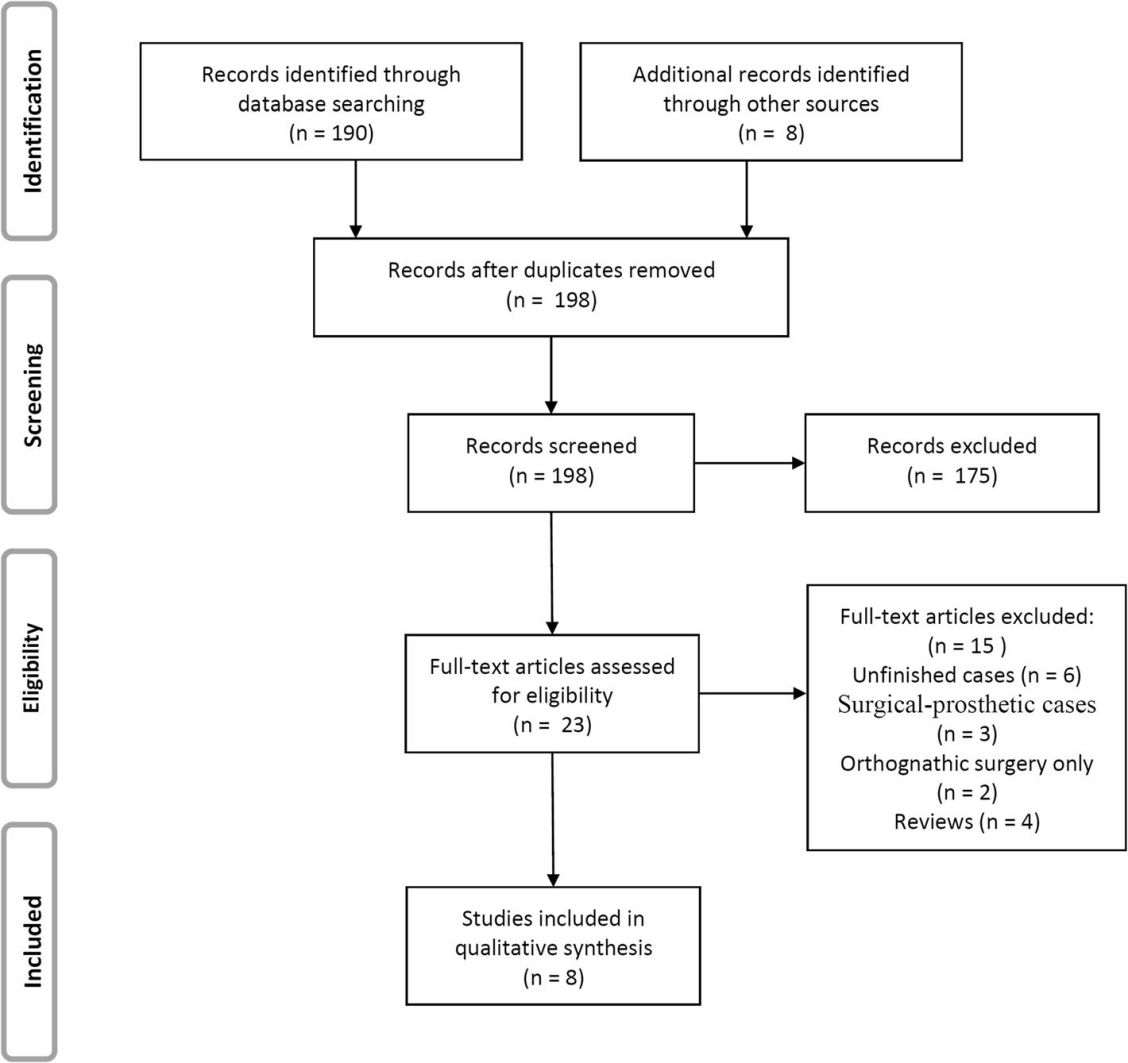
The flow diagram of literature search process

The search yielded a total of 190 primary papers from 3 electronic databases. After screening the literatures, 8 papers and 9 finished cases were included (inter-rater agreement = 91%, kappa = 0.87) [12–19]. The flow diagram of literature search process is presented in Fig. 7.

The treatment duration lasted from 2 years [12] to 13 years [13]. The age of patients ranged from 9 [14] to 28 years [15]. In terms of the treatment method, one included case observed for spontaneous eruption of impacted teeth which was similar to Toronto-Melbourne approach [14]. One used the method similar to Jerusalem approach [16]. Three cases facilitated the eruption of impacted teeth by orthodontic traction which was similar to Belfast-Hamburg approach [13, 15, 17] Another 4 cases were further treated with orthognathic surgery which were similar to Bronx approach [12, 18, 19]. The detailed characteristics and treatment approaches of all included cases were shown in Table 1.

**Table 1:** The detailed characteristics and treatment approaches of the included cases

First author	Year of publication	Country	Age	Sex	Treatment	Treatment duration
Frame [14]	1988	Britain	9	Male	Extraction of all primary and supernumerary teeth to allow the natural eruption. Thirteen permanent teeth partially or completely spontaneously erupted during observation. Orthodontic treatment.	4 years
Frohberg [19]	1995	America	11	Female	Sequentially extraction of the primary and supernumerary teeth Surgical exposure of the impacted permanent teeth for natural eruption. Combined orthognathic and orthodontic treatment.	Unknown
Angle [16]	2005	America	10	Female	Extraction of 10 supernumerary teeth. Surgical exposure of impacted incisors and upper canines with orthodontic traction. Exposure the entire clinical crowns of incisors by gingivectomy. Then surgical exposure of mandibular canines and all first premolars for natural eruption. Extraction of the mandibular second premolars and orthodontic treatment. Implants supported prostheses to replace 12 and 22.	8 years
Farronato [15]	2009	Italy	28	Male	Extraction of all primary and 6 supernumerary teeth. Surgical exposure of 11 impacted teeth with orthodontic traction. Orthodontic treatment. Crown restoration for 13–23 for esthetic motivation.	Unknown
Park [18]	2013	Asian	12	Male	Extraction of 9 primary teeth and 7 supernumerary teeth. Observation for possible spontaneous eruption of permanent teeth over 9 months. Surgical exposure combined with orthodontic traction of 32 and 42 which failed to erupt spontaneously. Combined orthognathic and orthodontic treatment. Implants supported prostheses to replace 12, 23, 33, and 43.	12 years
		Caucasian	14	Female	Extraction of all primary and supernumerary teeth. Maxillary expansion. Surgical exposure of 7 impacted teeth with orthodontic traction. Combined orthognathic and orthodontic treatment.	4 years
Rocha [13]	2014	Brazil	22	Female	Extraction of the 3 supernumerary teeth. Sequentially surgical exposure of 11 impacted teeth with orthodontic traction. Orthodontic treatment.	13 years
Cimen [12]	2015	Turkey	18	Male	Extraction of the 3 supernumerary teeth. Surgical exposure of all 10 impacted teeth with orthodontic traction under general anesthesia. Combined orthognathic and orthodontic treatment. Implants supported prostheses to replace 15–24.	2 years
Li [17]	2016	China	23	Male	Extraction of all primary and 9 supernumerary teeth. Surgical exposure of 4 impacted teeth with orthodontic traction. Orthodontic treatment. Implants supported prostheses to restore 45, 46.	Unknown

## Discussion

CCD is a complex congenital disease with skeletal anomalies and irregular dentition. The treatment plan greatly depends on patient’s demand, age, social and economic circumstances, eruption status of permanent dentition, periodontal and endodontic health. For young patients with a great demand of complete dentition and lasting oral function, a combined surgical-orthodontic treatment was proposed. Although the four surgical-orthodontic approaches mentioned above were well-known, the treatment methods varied from case to case.

Timing of the intervention is critical for CCD. Hitchin and Fairley proposed that the failure of eruption in CCD was due to lack of resorption of the overlying alveolar bone. The affected teeth would show a normal eruption pattern when they were uncovered [20]. Later on, Farrar and Van proposed the early surgical treatment with serial uncovering the impacted teeth [21]. Frame’s case reported the spontaneous eruption of the impacted teeth after clearing the way of eruption path in a 9-year-old boy, which proved Toronto-Melbourne approach [14]. The early intervention of the surgical exposure facilitated the spontaneous eruption of teeth and decreased the complexity of orthodontic treatment in the future.

As this patient started treatment at age of 16, missing the best period for spontaneous eruption, the approach advocated in this case was similar to the Belfast-Hamburg approach. Thus more surgeries and longer treatment duration might be required and more uncertainty might be added to the treatment results. Three of the included studies respectively reported successful cases treated by surgical exposure combined with orthodontic treatment similar to this patient [13, 15, 17]. Compared with the previous cases, this case was more challenging. A great number of impacted teeth accompanied by follicular cysts, the 7 mm crossbite, and the fact that right mandibular permanent premolars located so deep and so close to the inferior alveolar nerve added uncertainty to the orthodontic traction. The hypoplasia of the width of the maxilla and the skeletal discrepancy of upper and lower jaws increased the complexity of compensation treatment. The satisfying result should owe to the persistence of patient, the cooperation of surgeons and orthodontists, correct orientation of traction, and the carefully controlled orthodontic force. As the orthodontic traction and compensation treatment were time consuming, it was important to guarantee the periodontal and endodontic health of patients. The impacted anterior teeth were suggested to be exposed first for patients’ esthetics and self-esteem.

Three of included studies reported the cases treated with orthodontic and orthognathic treatment [12, 18, 19]. Except for two cases who were not yet of age to do orthognathic surgery delaying the treatment duration, 1 case finished the treatment in 2 years [12] and another case in 4 years [18]. Orthognathic surgery solves the skeletal deformity of CCD and spares the time of compensation treatment. So this approach can achieve the best treatment results with least time, but patients will have to suffer from the orthognathic surgery and the cost is high.

Prosthetic treatment is another treatment choice for patient with CCD. Atil and Petropoulos respectively reported cases describing oral rehabilitation with implant-supported fixed dental prostheses in middle-aged patients with CCD [22, 23]. Prosthetic treatment can rehabilitate oral esthetics and function in a short time and avoid sufferings from surgery and orthodontic treatment. However, this therapeutic method is more suitable for older patients. Adolescents tend to retain their own teeth and can’t accept being restored by prostheses which may have to be replaced several times during lifetime.

## Conclusions

In conclusion, CCD is a complex congenital disease with skeletal anomalies and irregular dentition. The treatment plan greatly depends on patient’s demand, age, economic circumstances, eruption status of permanent dentition, periodontal and endodontic health. Timing of treatment is important for CCD patients. Removing the primary and supernumerary teeth together with the bone covering the impacted teeth in early age can facilitate the spontaneous eruption of impacted teeth. For adults, combined surgical-orthodontic treatment can achieve a nearly complete dentition and stable occlusal contact, but it is time consuming and needs repeated surgeries. If possible, the combination of orthognathic surgery can reduce the difficulty of orthodontic treatment and treatment duration, as well as achieve a better facial profile.

## Abbreviation

CCD: Cleidocranial dysplasia
